# Removal of Cr (VI) from Simulated and Leachate Wastewaters by Bentonite-Supported Zero-Valent Iron Nanoparticles

**DOI:** 10.3390/ijerph15102162

**Published:** 2018-10-01

**Authors:** Fayuan Wang, Weiwei Yang, Fangyuan Zheng, Yuhuan Sun

**Affiliations:** College of Environment and Safety Engineering, Qingdao University of Science and Technology, Qingdao 266042, China; yangww3952@163.com (W.Y.); zhengfy9890@163.com (F.Z.)

**Keywords:** bentonite, zero-valent iron, chromium pollution, removal percentage, nanoparticles

## Abstract

Zero-valent iron (Fe^0^) nanoparticles (NPs) have shown excellent ability to remove contaminants hexavalent chromium (Cr(VI)) from aquatic systems. Use of support materials can help to prevent oxidation and aggregation of Fe^0^NPs, and thus enhance their remediation efficiency. However, most previous studies were conducted using artificially synthetic wastewater, and little is known on the remediation effects of supported Fe^0^NPs on actual wastewaters containing Cr(VI). Here, bentonite-supported Fe^0^NPs (BFe^0^NPs) with 1–5% of bentonite were prepared and characterized using scanning electron microscopy (SEM) and X-ray diffraction (XRD) techniques. Batch experiments were performed to study Cr(VI) removal by the selected BFe^0^NPs from a simulated wastewater and a leachate wastewater originating from a Cr slag heap-polluted soil. The results show that Fe^0^NPs were uniformly dispersed on the bentonite, leading to a decreased aggregation of NPs, and the optimal mass ratio of bentonite was 4%. Batch experiment results show that lower pH values favored Cr(VI) removal by BFe^0^NPs. The removal percentage of Cr(VI) was higher than 90% for both wastewaters when the pH value was 2.0, but decreased significantly as pH value increased. Cr(VI) removal reaction was quite fast within the initial 10 min, and at least 85% of Cr(VI) was removed for both wastewaters. Cr(VI) removal percentage increased with increasing BFe^0^NPs dosages ranging from 30 to 60, but remained almost unchanged when the Fe/Cr mass ratio increased to above 60. The reaction of BFe^0^NPs to remove Cr(VI) followed the pseudo second-order reaction model. In most cases, the removal rates of Cr(VI) were higher in simulated wastewater than in leachate wastewater, but all approached 100% at the optimal conditions. Our present results show that BFe^0^NPs with 4% bentonite are efficient for treatment of Cr(VI)-containing wastewaters.

## 1. Introduction

The compounds containing chromium (Cr) are among the most common pollutants in soil and groundwater [1,2]. Cr(III) and Cr(VI) are two main fractions in Cr-containing compounds. Cr(VI) is generally more toxic than Cr(III) for both acute and chronic exposures [3]. Cr(VI) can enter human bodies via ingestion, inhalation, or even directly from skin and move to organs such as liver, kidney, and lung, inducing genotoxicity, carcinogenicity, mutagenicity, and teratogenicity [4,5]. Cr(VI) compounds have been classified as a class I carcinogen for humans by IARC and listed as one of the priority contaminants [6]. Therefore, effective techniques need to be developed for remediation of Cr(VI)-contaminated soils and groundwater.

Numerous studies have shown that zero-valent iron (Fe^0^) can be used for remediation of pollutants including Cr(VI) in groundwater and wastewater [7,8,9,10,11]. In recent years, due to the larger specific surface area and higher reactivity than common Fe^0^, Fe^0^ nanoparticles (NPs) have shown promising potential in reduction of Cr(VI) [8,11]. However, due to their smaller particles with larger surface energy and intrinsic magnetic interactions, Fe^0^NPs readily become oxidized and agglomerated, leading to a low remediation efficiency. To prevent the agglomeration and oxidation of Fe^0^NPs, various support materials have been introduced, such as polymer resin [12], Gum Karaya [13], bentonite [14,15,16], surfactant-modified zeolite [17], montmorillonite [18], pumice [19], kaolinite [20], sepiolite [21], chitosan [22,23], carbon [24,25], biochar [26,27,28], humus [29], fly ash-based adsorbent [30], tetraethyl orthosilicate and hexadecyltrimethoxysilane [31], layered double hydroxide [32], graphite [33], titanate nanotube [34], magnetic Fe_3_O_4_/graphene nanocomposites [35], and reduced graphene oxide-alginate beads [36], most of which have shown enhanced reaction activity and removal rates of Cr(VI).

Among the studied support materials, bentonite is one of the low-cost and efficient adsorbents. Bentonite is a common clay mineral, consisting mainly of montmorillonite with a high cation exchange capacity and a large specific surface area, and can be used as an efficient adsorbent for removing toxic metals from wastewaters [37]. Bentonite-supported Fe^0^NPs with 1:1 mass ratio of iron and bentonite have been shown to significantly decrease their aggregation and to increase Fe^0^NPs reactivity, thus producing an enhanced Cr(VI) removal efficiency [16], which also varies with the factors such as initial content of Cr(VI), pH, and BFe^0^NPs dose [14,15]. However, because of the negative-charged surface of montmorillonite and the oxo-anionic form of hexavalent chromates in wastewater, bentonite generally has a poor ability to adsorb Cr(VI) from aqueous solution [16]. Therefore, a higher bentonite content may not increase or even inhibit the removal of Cr(VI) by BFe^0^NPs. In addition, most previous studies used artificially simulated Cr(VI) wastewater, but co-existing ions and organic materials affected Cr(VI) removal efficiency [38]. Thus far, little is known on whether Fe^0^NPs can effectively remediate actual wastewater and polluted underground water. It is of significance to expand the application fields of Fe^0^NPs.

Cr slag pollution poses great environmental risks in China [39]. High levels of Cr(VI) occur in Cr slag heap-polluted sites, causing serious pollution to soil, groundwater and surface water. It is of great significance to investigate the use of Fe^0^NPs to remediate Cr(VI) wastewater. In our current experiments, BFe^0^NPs with 1–5% bentonite were synthesized and characterized, and both simulated Cr(VI) wastewater and actual leachate wastewater from a Cr slag polluted site were used as targets. Our aims are: (1) to study the factors influencing Cr(VI) removal by BFe^0^NPs with a low bentonite content using batch experiment; and (2) to investigate the utility of BFe^0^NPs for Cr(VI) removal from simulated and actual Cr(VI) wastewaters.

## 2. Materials and Methods

### 2.1. Materials and Reagents

The bentonite was primarily sodium montmorillonite (Na-Mt) purchased from Shanghai No. 4 Reagent & H. V. Chemical Co. Ltd. (Shanghai, China), with a montmorillonite content >90%. The particle size of the bentonite is <37 μm. The composition of the bentonite is SiO_2_ (61.64%), Al_2_O_3_ (17.08%), Fe_2_O_3_ (3.95%), CaO (1.71%), MgO (2.78%), K_2_O (0.86%), and Na_2_O (4.33%). FeSO_4_ and NaBH_4_ were purchased from Sinopharm Chemical Reagent Co. Ltd. (Shanghai, China). K_2_Cr_2_O_7_ was purchased from Tianjin Standard Technology Co. Ltd. (Tianjin, China). All chemicals were analytical-reagent grade.

### 2.2. Synthesis of BFe^0^NPs

Briefly, 5.56 g FeSO_4_·7H_2_O was added into a 250 mL conical flask with 100 mL distilled water, and stirred with a magnetic stirrer until all powder was dissolved. A certain quantity of bentonite was added to the above solution to obtain BFe^0^NPs with different percentages of bentonite. After stirring for 30 min with a magnetic stirrer, the mixture was centrifuged at 10,000 rpm, and the precipitated solids were separated and transferred to 250 mL conical flasks. NaBH_4_ solution prepared by dissolving 1.5 g NaBH_4_ in 50 mL of deionized water was added dropwise into the flasks. Black solid Fe^0^ particles were observed immediately after the first drop of NaBH_4_ solution was added. After all the NaBH_4_ solution was added, the mixture was kept for another 30 min of stirring. The reduction between iron and borohydride can be indicated with the following reaction:

4Fe^2+^ + 3BH_4_^−^ + 9H_2_O ➝ 4Fe^0^ + 3H_2_BO_3_^−^ + 8H^+^ + 8H_2_

The solid particles were separated magnetically, and quickly washed triple times with pure ethanol, and then oven-dried at 60 °C overnight. Through changing the mass ratio, the BFe^0^NPs with bentonite at 1%, 2%, 3%, 4%, and 5% were synthesized following the same procedure. Pre-experiments have found that BFe^0^NPs with a bentonite content of 4% has the largest removal percentage of Cr(VI) from wastewater, and thus they were selected in the following batch experiments.

### 2.3. Characterizations and Measurements

To characterize the overall size distribution and morphology of BFe^0^NPs, scanning electron microscope (SEM) (6700F, JEOL, Tokyo, Japan) equipped with X-ray energy dispersive spectrometer (EDS) was used. SEM images were taken at different magnifications at an operating voltage of 30 kV.

Characterization of BFe^0^NPs was performed using an X-ray diffractometer (XRD, Rigaku D/max-2500 PC, Rigaku Industrial Corp., Tokyo, Japan), operating with Cu Kα radiation at 40 kV and 150 mA, scanning over the range 5°–90° in 2*θ*, step size 0.02°.

The concentration Cr (VI) in solution was detected using the 1,5-diphenylcarbazide method with a visible spectrophotometer (TAS-986, Shanghai Lengguang Technology Co. Ltd., Shanghai, China).

### 2.4. Simulated Wastewater and Actual Wastewater

Simulated wastewater containing 50 mg/L Cr(VI) was obtained by dissolving K_2_Cr_2_O_7_ in deionized water. Cr-polluted soil was collected from a Cr slag heap site at Qingdao Hongxing Chemical Co. Ltd. (Qingdao, China). To investigate the applicability to remove Cr(VI) from actual wastewater, the leachate was prepared by washing 200 g Cr-polluted soil using 800 mL 0.5 M HCl. The wastewater was centrifuged and then filtered, with the following characteristics, pH 2.1, Cr(VI) 52.89 mg/L, Fe 586.19 mg/L, Mn 49.01 mg/L, Ca 1544.09 mg/L, and Mg 239.30 mg/L.

### 2.5. Batch Experiments

Batch experiments were conducted at room temperature (25 °C) on a mechanical shaker at 120 rpm using 100 mL conical flasks. The wastewater was sampled after shaking and filtered through 0.45 μm filter membrane for analysis of Cr(VI).

#### 2.5.1. Effect of the pH Value

The pH values of Cr(VI) wastewater were adjusted with HCl (0.1 M) or NaOH (0.1 M) solutions. Then, 60 mg BFe^0^NPs were added into the flasks with 20 mL simulated or actual wastewaters, and then agitated at 25 °C for 2 h at 120 rpm.

#### 2.5.2. Effect of Reaction Time

The second experiment was to determine the impact of reaction time and equilibrium time. The pH of Cr(VI) wastewater was adjusted to 2.0, 2.5, and 3.0, respectively. Wastewater (20 mL) and 60 mg BFe^0^NPs were added into the flasks and then agitated at 120 rpm for reaction times ranging from 10 to 180 min. Samples were taken at 10, 20, 30, 45, 60, 90, 120, and 180 min, respectively.

#### 2.5.3. Effect of BFe^0^NPs Dosage

BFe^0^NPs at different doses, i.e., 30, 45, 50, 60, 70, 100, and 150 mg, were added into the flasks containing 20 mL simulated or actual wastewaters at pH 2.0, and then agitated at 25 °C for 2 h at 120 rpm.

### 2.6. Calculation of Removal Percentage and Removal Capacity

Cr(VI) removal percentage (%), the capacity of Cr(VI) removal per unit mass of BFe^0^NPs at time *t* (*q*_t_, mg/g), and the capacity of Cr(VI) removal per unit mass of BFe^0^NPs at equilibrium (*q*_e_, mg/g), were calculated from the following equations [40]:

(1) $$\mathrm{Cr}\left(\mathrm{VI}\right)\;\mathrm{removal}\;\mathrm{percentage}\;=\;\frac{C_{0}-C_{e}}{C_{0}}\;\times \;100$$

(2) $$qt\;=\;V\;\times \;\frac{C_{0}-C_{t}}{m_{s}}$$

(3) $$qe\;=\;V\;\times \;\frac{C_{0}-C_{e}}{m_{s}}$$

where *C*_0_ and *C*_e_ (mg/L) are the initial and the final Cr(VI) concentrations in the wastewaters in flasks, respectively, and *C*_t_ (mg/L) is the Cr(VI) concentrations at time *t*. V is the volume of wastewater (L) and *m*_s_ is the mass of BFe^0^NPs added (g).

### 2.7. Kinetics of Cr(VI) Removal by BFe^0^NPs

The kinetics of Cr(VI) were tested using pseudo-second-order sorption equation [40]:

(4) $$\frac{t}{qt}\;=\;\frac{1}{{kqe}^{2}}\;+\;\frac{t}{qe}$$

where *k* [mg·(mg·min)^−1^] is the rate constant of the pseudo-second-order reaction.

### 2.8. Data Analysis

The nanoparticle size distribution of Fe^0^ was determined by Nano Measurer 1.2 software (Beijing Zhongke Baice Technology Service Co. Ltd., Beijing, China). The XRD data were analyzed using Jade 7.1.2 software (Jade Software Corp., Christchurch, New Zealand). The data were processed with Excel 2010 (Microsoft Corp., Redmond, WA, USA) and Origin 6.0 software (OriginLab, Northampton, MA, USA). Means with standard deviations of Cr(VI) removal percentage, *q*_t_, and *q*_e_ were calculated. Linear regression analysis was performed to calculate reaction kinetics and the reaction rate constant.

## 3. Results and Discussion

### 3.1. Characterization of BFe^0^NPs

**Figure 1:**
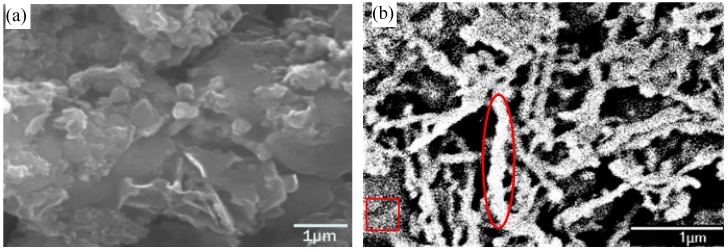

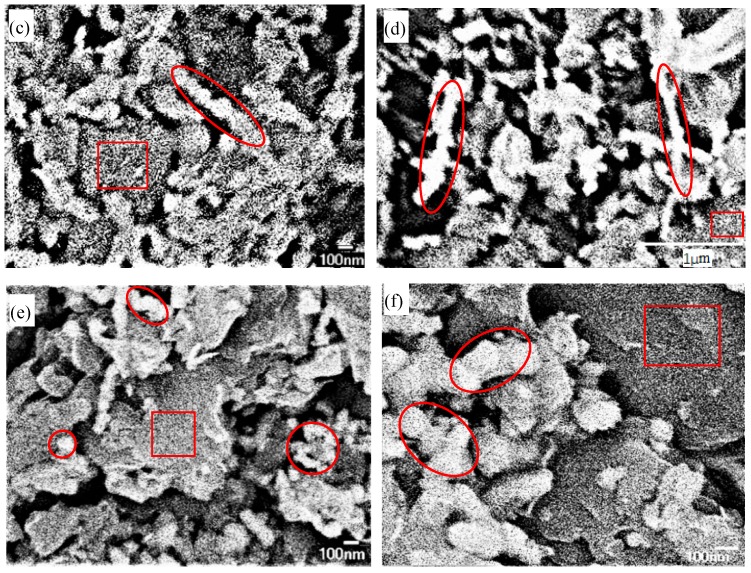
SEM images of bentonite (**a**) and BFe^0^NPs with different bentonite contents (**b**–**f**): (**a**) bentonite; (**b**) 1%; (**c**) 2%; (**d**) 3%; (**e**) 4%; and (**f**) 5%. The white chain-like conformations and dispersed particles represent Fe^0^ (circles/ovals), and the grey backgrounds represent bentonite (rectangles).

The morphology of bentonite and distribution of Fe^0^NPs on bentonite were investigated using SEM (Figure 1). The pure bentonite displayed an anomalous layer structure with a relatively smooth surface. When loaded on the bentonite, Fe^0^NPs were found to disperse on the surface and edges of bentonite (Figure 1b–f). The particle size of Fe^0^NPs dispersed on bentonite varied in the range 50–120 nm, with an average of about 77 nm. Some Fe^0^NPs on the bentonite appeared to aggregate into chain-like conformations, however, the degree of aggregation decreased with the increase of the mass ratio of bentonite, and the BFe^0^NPs with 4% and 5% of bentonite appeared to have less chain-like conformations than other BFe^0^NPs with less bentonite. The aggregated proportion of Fe^0^NPs tended to decline as the Fe(II) precursor concentration decreased [14,16].

Using a bentonite/iron mass ratio of 1:1, Shi et al. [14,16] also found decreased aggregation of Fe^0^ particles on the bentonite. Bentonite generally shows a poor adsorption capacity for Cr(VI)-containing compounds [16]. Hence, Cr(VI) removal by BFe^0^NPs is mainly attributed to the reduction capacity of Fe^0^. Our results show that 4% of bentonite is effective to reduce aggregation of Fe^0^NPs and to enhance the stability of Fe^0^NPs. A comparison pre-experiment confirmed BFe^0^NPs with 4% of bentonite had the highest Cr(VI) removal capacity (data not shown).

**Figure 2:**
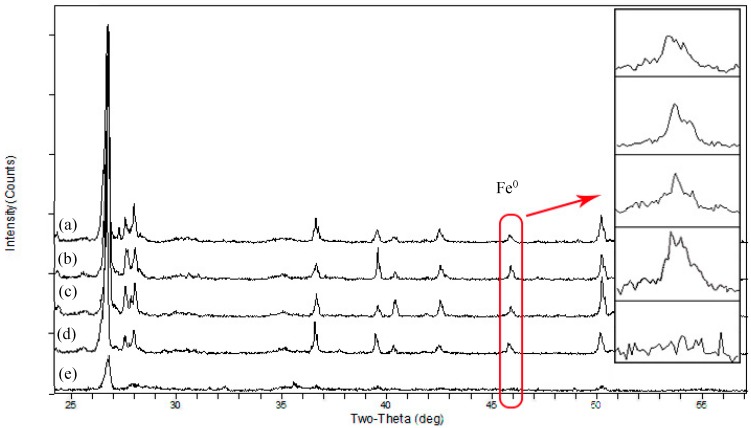
XRD characterization of BFe^0^NPs with different bentonite contents: (**a**) 1%; (**b**) 2%; (**c**) 3%; (**d**) 4%; and (**e**) 5%.

The XRD patterns of BFe^0^NPs with different bentonite contents showed a number of peaks. The largest peak (2*θ* = 26.67°) and other smaller peaks stem from the internal structures of bentonite (Figure 2). The characteristic peaks of Fe^0^ (2*θ* = 45.8°) were observed, which was stronger in BFe^0^NPs with 4% bentonite than in BFe^0^NPs with other contents of bentonite (Figure 2). When the bentonite content is 5%, more Fe^0^NPs may enter into the inner layer of bentonite, thus leading to a weak peak. This confirms the mass ratio of Fe and bentonite influences the dispersity and stability of Fe^0^ in the NPs. An optimal mass ratio should be selected to ensure not only the “support” role of bentonite, but also the reactivity of Fe^0^.

### 3.2. Factors Influencing Cr(VI) Removal

#### 3.2.1. Effect of Wastewater pH

**Figure 3:**
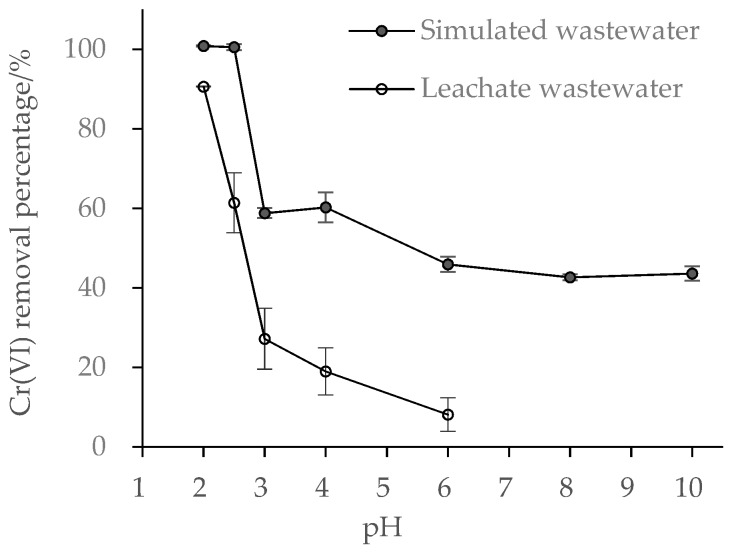
Effect of initial pH values on Cr (VI) removal (*n* = 3) from wastewaters by BFe^0^NPs (4%).

The solution pH is among the most significant factors that determine both the forms of metal ion and the surface characteristics of the adsorbent in the solution [20]. In aquatic solution, Cr(VI) occurs mainly as HCrO_4_^−^ or Cr_2_O_7_^2−^ at pH 1.0 to 6.0, and as CrO_4_^2−^ over pH 6.0 [2]. A previous study has shown that Cr(VI) removal rate reached 100% within 1 min at pH 2.0, but was lower than 30% at pH 8.0 [14]. Here, simulated wastewater was adjusted to pH 2.0–10.0, while leachate wastewater was adjusted to pH 2.0–6.0 based on the results obtained from simulated wastewater. Our results show similar conclusions that Cr(VI) removal decreased sharply from 100% to 60% for simulated wastewater and from 90% to 28% for leachate wastewater when the pH increased from 2.0 to 3.0 (Figure 3). At pH 6.0, only 50% of Cr(VI) was removed from simulated wastewater and 10% from leachate wastewater. At pH 8.0, 45% of Cr(VI) was removed from simulated wastewater, higher than that reported by Shi et al. [14], which can be attributed to the higher content of Fe^0^ in the BFe^0^NPs.

Our findings confirm the similar conclusion obtained by Shi et al. [14] that acidic pH enhanced Cr(VI) removal. At lower pH, both HCrO_4_^−^ and Cr_2_O_7_^2−^ anions are strong oxidizers with high redox potentials, whilst CrO_4_^2−^ anion existing at high pH has only a weak oxidizing capacity [9]. Apparently, redox reaction occurs more readily between Fe^0^NPs and Cr(VI) at lower pH. Another possible reason is a lower pH accelerates the corrosion of Fe^0^NPs, and Cr(III) and Fe(III) hydroxides cannot easily precipitate on the NPs surfaces, thus leading to an increased reaction rate [41]. However, at a higher pH, co-precipitates of Cr(III) and Fe(III) on the surface of BFe^0^NPs may hinder the reduction of Cr(VI) by Fe^0^. Furthermore, increased H^+^ will neutralize the negative charge on the surface of bentonite and reduce the electrostatic repulsion against anionic Cr(VI). This consequently leads to enhanced redox reaction of Fe^0^ and Cr(VI) [42].

Another finding is that Cr(VI) removal percentage was always lower in leachate wastewater than in simulated wastewater (Figure 3). Cr(VI) reduction by Fe^0^ varies with experimental conditions including solution composition [9]. Previous studies have shown that the remediation effects of Fe^0^ are positively or negatively influenced by co-existing ions such as anionic PO_4_^3−^, SiO_3_^2−^, SO_4_^2−^, NO_3_^−^, HCO_3_^−^, and Cl^−^; cationic Ca^2+^, Mg^2+^, Na^+^, Zn^2+^, Cu^2+^, and Cd^2+^; and natural organic matter [38,43,44,45]. For example, most of the anions especially HCO_3_^−^ significantly suppressed Cr(VI) removal, but cations and natural organic matter enhanced the removal [38]. In Cr(VI)-polluted soil, humic acid facilitated the reduction of Cr(VI) [46]. In our experiments, simulated wastewater was pure solution of K_2_Cr_2_O_7_, while leachate wastewater originated from Cr-polluted soil containing more complex components in addition to Cr(VI), such as Fe^3+^, Mn^2+^, Ca^2+^, Mg^2+^, other soluble anions, and natural organic matters. These materials especially oxidized ions may compete with Cr(VI) for reaction with Fe^0^, and occupy the adsorption sites on the surface of bentonite, thus resulting in a lower Cr(VI) removal percentage.

However, this finding differs from a previous study that 100% of total Cr was removed by BFe^0^NPs from an electroplating wastewater, and the concomitant ions Cu, Pb, and Zn did not impact Cr removal percentage [14]. Electroplating wastewaters are generally high in heavy metals, such as Cr(VI), Pb^2+^, Cu^2+^, and Zn^2+^, but low in organic matter, while in our present study, leachate wastewater may contain different reductive materials such as dissolved organic matter, in addition to Mn^2+^, which may explain the lower Cr(VI) reduction by BFe^0^NPs.

#### 3.2.2. Effect of Reaction Time

**Figure 4:**
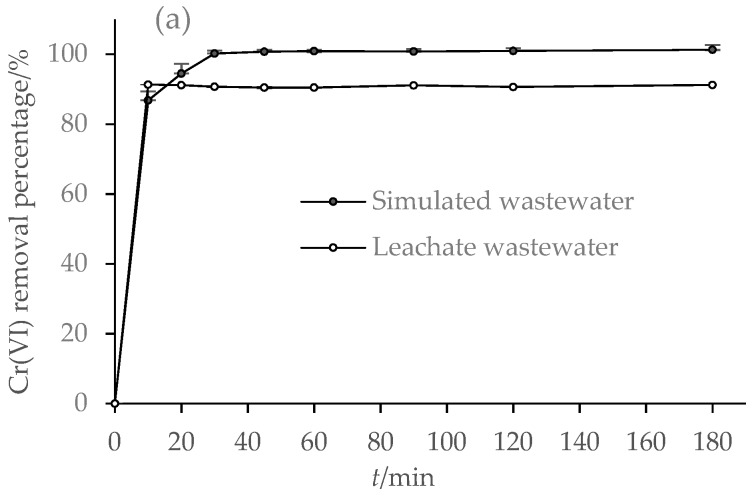

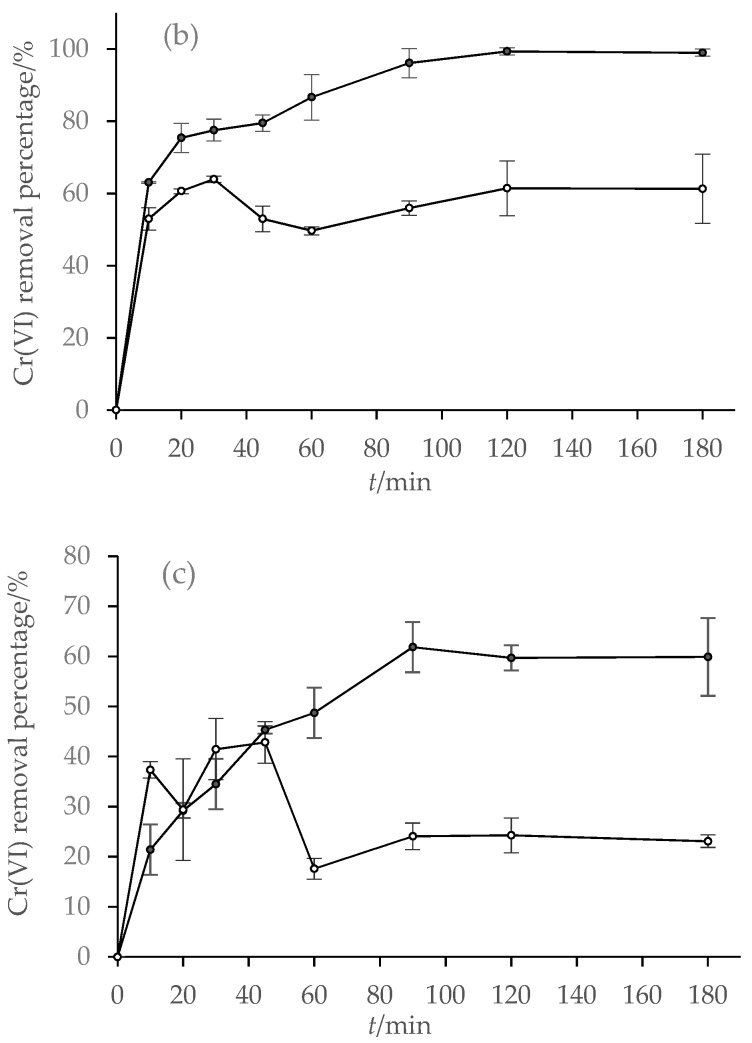
Effect of reaction time on Cr (VI) removal (*n* = 3) from wastewaters by BFe^0^NPs (4%) at different pH values: (**a**) pH 2.0; (**b**) pH 2.5; and (**c**) pH 3.0.

Figure 4 shows Cr(VI) removal percentages of two wastewaters at pH 2.0 varying with different reaction times. Similarly, as shown in Figure 3, Cr(VI) removal percentages were lower in leachate wastewater than in simulated wastewater. However, Cr(VI) removal was very fast initially for both two wastewaters; about 85% of Cr(VI) was removed from simulated wastewater within 10 min, and Cr(VI) was completely removed within 60 min, indicating an equilibrium time of 60 min at pH 2.0. For leachate wastewater, Cr(VI) removal percentage reached 90% within the initial 10 min, and maintained at about 90% thereafter. However, at pH 2.5 and 3.0, equilibrium times increased to 120 min, and Cr(VI) removal percentages decreased significantly especially for leachate wastewater.

The high Cr(VI) removal and short equilibrium time confirm that bentonite was effective to maintain dispersity and activity of Fe^0^ particles. Obviously, at the initial stage, redox reaction was fast because of the abundance of Cr(VI) and active Fe^0^ particles. As reaction time prolonged, the amount of active Fe^0^NPs became less, and, thus, the redox reaction became more difficult. In addition, precipitations of reaction products such as Cr(III) and Fe(III) may also occupy the active sites of BFe^0^NPs and hinder the redox reaction [41].

As shown in Figure 4, Cr(VI) removal increased sharply within the first 10 min, and then rose slightly or even decreased in the later reaction process, suggesting the removal of Cr(VI) by BFe^0^NPs is more than a simple chemical reaction [9,14]. Previous studies have shown that other physical reactions such as adsorption phase probably occur [12,14,47]. In our current experiment, a physical mechanism such as reversible adsorption may also occur, especially before reaching equilibrium. Furthermore, co-existing materials in leachate wastewater may disturb Cr(VI) reduction or compete with the adsorption of Cr(VI). The different trends for Cr(VI) removal from the two wastewaters suggest that the overall mechanisms are much complicated in leachate wastewater than in simulated wastewater.

#### 3.2.3. Effect of BFe^0^NPs Dosage

**Figure 5:**
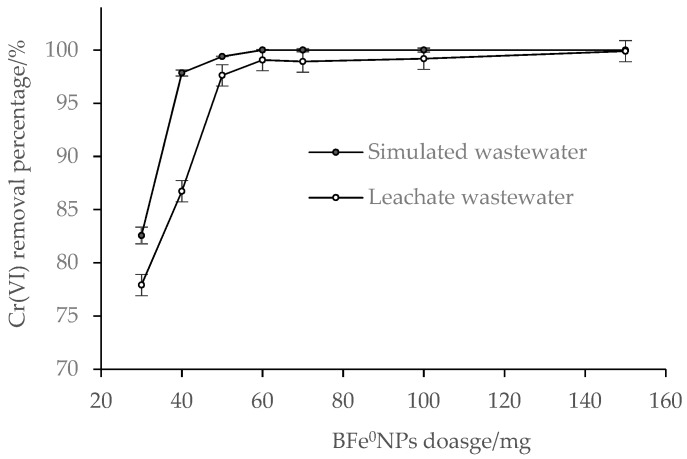
Effect of BFe^0^NPs (4%) dosage on Cr (VI) removal (*n* = 3) from wastewaters.

The Cr (VI) removal from simulated wastewater increased rapidly as the BFe^0^NPs dosage increased from 30–40 mg, and slowly from 40–60 mg, but remained almost unchanged ranging from 60 to 150 mg (Figure 5). Comparatively, Cr(VI) removal from leachate wastewater increased dramatically as the BFe^0^NPs dosage increased from 30 to 60 mg, and thereafter, increased slowly from 60 to 150 mg. Obviously, the increase in Cr(VI) removal with increasing BFe^0^NPs dosage can be attributed to the increased active sites, where the reduction takes place [23,42]. When the BFe^0^NPs reach an equilibrium dosage providing enough reactive sites for Cr(VI) reduction, Cr(VI) removal will maintain constant, and not increase with the increasing dosage of BFe^0^NPs.

Overall, Cr(VI) removal percentage was lower in simulated wastewater than in leachate wastewater, especially at the low dosage of BFe^0^NPs, but the difference became less with the increase of BFe^0^NPs dosage, and no difference was observed when 150 mg BFe0NPs were added. This can be attributed to the co-existing materials in leachate wastewater competing with Cr(VI) for active sites in Fe^0^NPs and/or adsorption sites in bentonite. When BFe^0^NPs dosage was high enough, the impacts of the co-exiting materials were offset, and therefore nearly 100% of Cr(VI) was removed from leachate wastewater.

In Figure 5, a dosage of 60 mg BFe^0^NPs may be effective and economic for their actual application in remediation of Cr(VI) wastewater. Regardless of the cost, a higher dosage may cause aggregation of BFe^0^NPs, thus leading to a lower reaction efficiency and Cr(VI) removal capacity.

### 3.3. Reaction Kinetics

**Figure 6:**
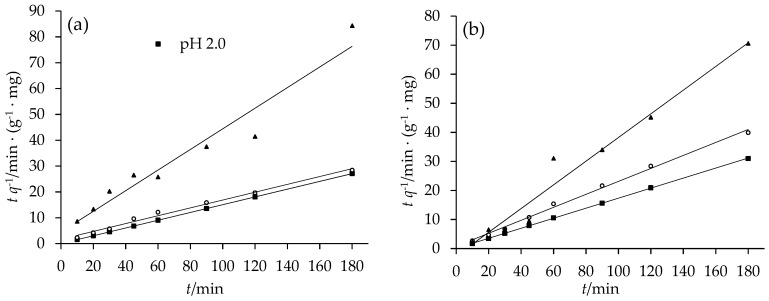
Pseudo second-order model for Cr(VI) removal by BFe^0^NPs (4%) at different pH values: (**a**) simulated wastewater; and (**b**) leachate wastewater.

The reaction kinetics were investigated using wastewaters at different pH values in the reaction time of 180 min. Kinetic parameters including second order rate constant *k*, experimental equilibrium removal capacity *q*_e,exp_, calculated equilibrium removal capacity *q*_e,cal_, and regression coefficients (*R*^2^), are shown in Table 1. The *q*_e,cal_ values using the pseudo second-order model are in good accordance with the experimental values (*q*_e,exp_). The *R*^2^ values range from 0.9416 to 1, indicating that the system under study well fits the pseudo-second-order model (Figure 6). The good consistency between *q*_e,cal_ and *q*_e,exp_, together with the large *R*^2^ values, suggests the reaction process may involve in a chemical sorption [48], or valency forces through sharing or the electron exchange between Cr(VI) and BFe^0^NPs as covalent forces [49].

Through comparing *q*_e,cal_ and *q*_e,exp_, and *R*^2^ values at different pH values, our present results confirm the above-mentioned result (see Section 3.2.1) that acidic pH is good for Cr(VI) removal by BFe^0^NPs, which is consistent with previous results [14,16,23]. Although pH 3.0 also belongs to acidic condition, the Cr(VI) removal percentage remarkably decreased at this pH level. Moreover, the observed rate constant (*k*) decreased dramatically with the increasing initial pH, revealing the determinative role of solution pH in reaction between Cr(VI) and BFe^0^NPs.

Kinetics of reaction between Cr(VI) and Fe^0^ in aquatic systems are generally described by pseudo first-order, first-order, zero-order, or even reaction order less than unity (see review by Gheju [9]). However, pseudo second-order models have been found to best describe both Cr(VI) reduction with Fe@Fe_2_O_3_ core–shell nanowires [50], and the adsorption of As by montmorillonite-supported Fe^0^NPs [18]. Obviously, the results depend on the experimental conditions. Gould [51] studied Cr(VI) reduction with Fe^0^ over a variety of conditions, and found that Cr(VI) removal varied with the contents of Cr(VI) and H^+^, as well as specific surface area of Fe^0^. Our present findings on Cr(VI) reduction kinetics differ from Shi et al. [14,16], who found the removal of Cr (VI) by BFe^0^NPs fitted pseudo first-order reaction kinetics. The possible reason may be the bentonite/iron mass ratio (and the specific surface area). Shi et al. [14,16] used BFe^0^NPs with a 50% bentonite mass fraction, but, in our study, the bentonite content was only 4%. Furthermore, in the present study, leachate wastewater always showed lower Cr(VI) removal percentages than simulated wastewater, indicating there may be other factors influencing Cr(VI) reduction kinetics.

The main mechanisms of Cr(VI) reduction involve the capacity of Fe^0^ particles to serve as electron donor, as well as of Fe(II) generated as products of Fe^0^ particles corrosion [9]. Using XPS analysis, Zhang et al. [52] concluded Cr(VI) removal mechanisms by Fe^0^NPs as: (i) large surface area; (ii) a great number of active reaction sites; and (iii) fast electron transport from Fe^0^/FeCr_2_O_4_ to Cr(VI). BFe^0^NPs with a high mass ratio of Fe^0^ can provide more effective electron donors and active sites, compared to the same amount of BFe^0^NPs with a high content of bentonite. Furthermore, as the specific surface areas are generally higher for Fe^0^NPs than for bentonite [14], a high ratio of bentonite in BFe^0^NPs may lead to a low adsorption capacity. Taking these into account, BFe^0^NPs with 4% bentonite are applicable to eliminate Cr(VI) from both simulated wastewater and leachate wastewater. Undoubtedly, since the chemical compositions of actual wastewaters and polluted groundwater are generally complex and vary with environmental conditions, more attempts are needed to examine the applicability of support Fe^0^NPs for future remediation of Cr(VI) in aquatic systems under complex realistic conditions.

## 4. Conclusions

BFe^0^NPs with different bentonite contents were synthesized and characterized using SEM and XRD techniques. Fe^0^ particles at nano-scales were found to disperse on the bentonite, which probably leads to less aggregation and greater stability of Fe^0^NPs. Batch experiments were carried out to study Cr(VI) removal from two wastewaters by the selected BFe^0^NPs with 4% of bentonite at different pH values, reaction times, and BFe^0^NPs dosage levels. Results show that a low solution pH value (2.0) favored Cr(VI) removal. Cr(VI) was 100% removed from simulated wastewater and 90% from leachate wastewater when the pH value was 2.0, but decreased significantly with increasing pH value. The Cr(VI) removal by BFe^0^NPs was very fast initially, and at least 85% of Cr(VI) was removed within 10 min. Increase in BFe^0^NPs dosage led to increased Cr(VI) removal, and a dosage of 60 mg BFe^0^NPs was effective for remediation of Cr(VI) wastewater. Cr(VI) removal by BFe^0^NPs fitted the pseudo second-order kinetic model. The removal percentages of Cr(VI) by BFe^0^NPs were generally higher in simulated wastewater than in leachates wastewater, but all approached 100% at the optimal reaction conditions. Our results suggest that BFe^0^NPs with 4% bentonite could be effective for remediation of Cr(VI)-bearing wastewaters. Furthermore, more work needs to be conducted to better characterize the morphology of the nanostructures and to illustrate the mechanisms involved in Cr (VI) removal.

## Figures and Tables

**Table 1:** Pseudo second-order kinetic parameters for Cr(VI) removal from two wastewaters by BFe^0^NPs (4%) at different pH values.

pH	Simulated Wastewater				Leachate Wastewater			
	*q*_e,exp_/mg·g^−1^	*q*_e,cal_/mg·g^−1^	*k*/g (mg·min)^−1^	*R* ^2^	*q*_e,exp_/mg·g^−1^	*q_e,cal_*/mg·g^−1^	*k*/g (mg·min)^−1^	*R* ^2^
2.0	6.65	6.65	1.45	1	5.80	5.79	0.30	1
2.5	6.45	6.60	0.01	0.9907	4.68	4.48	0.06	0.9961
3.0	2.14	2.51	0.04	0.9416	2.55	2.45	−0.07	0.9664
